# Safety, immunogenicity and antibody persistence of a bivalent Beta-containing booster vaccine against COVID-19: a phase 2/3 trial

**DOI:** 10.1038/s41591-022-02031-7

**Published:** 2022-10-06

**Authors:** Spyros Chalkias, Frank Eder, Brandon Essink, Shishir Khetan, Biliana Nestorova, Jing Feng, Xing Chen, Ying Chang, Honghong Zhou, David Montefiori, Darin K. Edwards, Bethany Girard, Rolando Pajon, Frank J. Dutko, Brett Leav, Stephen R. Walsh, Lindsey R. Baden, Jacqueline M. Miller, Rituparna Das

**Affiliations:** 1 Moderna, Inc., Cambridge, MA USA; 2 Meridian Clinical Research, Binghamton, NY USA; 3 Meridian Clinical Research, Omaha, NE USA; 4 Meridian Clinical Research, Rockville, MD USA; 5 Department of Surgery and Duke Human Vaccine Institute, Durham, NC USA; 6 Brigham and Women’s Hospital, Boston, MA USA

**Keywords:** RNA vaccines, Viral infection

## Abstract

Updated immunization strategies are needed to address multiple severe acute respiratory syndrome coronavirus 2 (SARS-CoV-2) variants. Here we report interim results from an ongoing, open-label phase 2/3 trial evaluating the safety and immunogenicity of the bivalent Coronavirus Disease 2019 (COVID-19) vaccine candidate mRNA-1273.211, which contains equal mRNA amounts encoding the ancestral SARS-CoV-2 and Beta variant spike proteins, as 50-µg (*n* = 300) and 100-µg (*n* = 595) first booster doses administered approximately 8.7–9.7 months after the mRNA-1273 primary vaccine series (NCT04927065). The primary objectives were to evaluate the safety and reactogenicity of mRNA-1273.211 and to demonstrate non-inferior antibody responses compared to the mRNA-1273 100-µg primary series. Additionally, a pre-specified immunogenicity objective was to demonstrate superior antibody responses compared to the previously authorized mRNA-1273 50-µg booster. The mRNA-1273.211 booster doses (50-µg or 100-µg) 28 days after immunization elicited higher neutralizing antibody responses against the ancestral SARS-CoV-2 and Beta variant than those elicited 28 days after the second mRNA‑1273 dose of the primary series (NCT04470427). Antibody responses 28 days and 180 days after the 50-µg mRNA-1273.211 booster dose were also higher than those after a 50-µg mRNA-1273 booster dose (NCT04405076) against the ancestral SARS-CoV-2 and Beta, Omicron BA.1 and Delta variants, and all pre-specified immunogenicity objectives were met. The safety and reactogenicity profile of the bivalent mRNA-1273.211 booster (50-µg) was similar to the booster dose of mRNA-1273 (50-µg). Immunization with the primary series does not set a ceiling to the neutralizing antibody response, and a booster dose of the bivalent vaccine elicits a robust response with titers that are likely to be protective against COVID-19. These results indicate that bivalent booster vaccines can induce potent, durable and broad antibody responses against multiple variants, providing a new tool in response to emerging variants.

## Abstract (web summary)

A bivalent vaccine encoding the ancestral and Beta variant SARS-CoV-2 spike proteins boosts neutralizing antibody responses against multiple viral variants, suggesting that a bivalent approach is an effective strategy to broadly enhance protection against COVID-19.

## Main

The development and global deployment of SARS-CoV-2 vaccines has been important for reducing the burden of COVID-19. The mRNA-1273 vaccine encodes the spike protein of the ancestral SARS-CoV-2 (Wuhan-HU-1 isolate) and was well-tolerated and demonstrated 93.2% efficacy against COVID-19 after a median follow-up of 5.3 months after a two-dose 100-µg primary series in the phase 3 Coronavirus Efficacy (COVE) trial^1,2^.

SARS-CoV-2, similarly to other pathogenic coronaviruses, has the propensity to rapidly mutate, and many SARS-CoV-2 variants have emerged^3,4^. SARS-CoV-2 variants, such as Beta (B.1.351), have key antibody escape mutations in the spike protein, and others, such as the Delta variant (B.1.617.2), carry mutations associated with enhanced transmissibility^4^. The potential of Beta to circumvent immunity, and the association with increased morbidity and mortality, was not accompanied by a growth advantage, whereas the highly transmissible Delta variant became the dominant SARS-CoV-2 variant in multiple geographies in July–December 2021. In November 2021, the Omicron variant (B.1.1.529) emerged as the most antigenically divergent variant to date, with more than 30 mutations in the spike protein, 15 of which are clustered in the receptor-binding domain^5^. Omicron shares key antibody escape site mutations with the Beta variant, and it also exhibits substantial transmissibility advantages^6–8^.

During the placebo-controlled part of the COVE trial, the predominant circulating variants were the ancestral SARS-CoV-2 with the D614G mutation and the Alpha (B.1.1.7) variant; subsequent infection waves caused by other SARS-CoV-2 variants (Delta and Omicron) led to the need for booster doses^9–14^. After a 50-µg booster dose of mRNA-1273, neutralizing antibodies against variants such as Delta and Omicron were detectable at higher titers than after the mRNA-1273 primary series^10,15^. However, antibody titers, especially against antigenically divergent variants such as Omicron, appear to be lower than those against ancestral SARS-CoV-2 and wane over time after a 50-µg dose of the prototype booster^10^. In addition, emerging vaccine effectiveness data suggest decreased long-term booster vaccine effectiveness against infection symptomatic from Omicron, although protection against severe disease and hospitalization is maintained^16,17^.

When Beta first emerged in late 2020, Beta-specific neutralizing antibody titers after the primary series vaccination were found to be lower than the antibody titers against the ancestral virus^18^. It was hypothesized that a bivalent booster vaccine that contained mRNAs for the ancestral SARS-CoV-2 and the Beta spike protein could enhance the immune response by increasing antibody diversity^19^. mRNA-1273.211 contains equal amounts of two spike protein mRNA sequences—one for the ancestral SARS-CoV-2 and the other for the Beta variant—and it was the first modified, bivalent booster candidate to be evaluated in the clinic in a phase 2/3 booster vaccine study in adults. This study was designed to evaluate the safety, immunogenicity and durability of the antibody response to variant-matched booster vaccines. Here we summarize the safety and immunogenicity results of an interim analysis of two dose levels (50 µg and 100 µg) of the booster vaccine candidate mRNA-1273.211 to provide key safety and immunogenicity insights for bivalent booster vaccines. At present, Omicron-containing bivalent vaccines against COVID-19 are being used in multiple geographies^20^.

## Results

### Trial populations

**Fig. 1:**
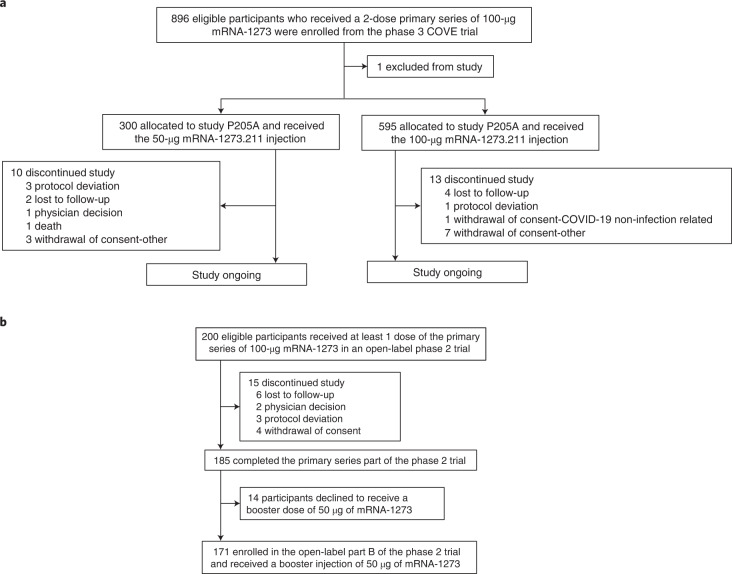
CONSORT (trial profile). **a**, Study P205: trial profile of participants in study P205 part A who received a booster dose of 50-µg mRNA-1273.211 or 100-µg mRNA-1273.211. **b**, Trial profile of participants who received a 50-µg booster dose of mRNA-1273 in part B of the phase 2 trial.

From 28 May to 4 June 2021 and from 30 June to 16 July 2021, a total of 895 participants from the phase 3 COVE trial received a single booster dose of 50 µg (*n* = 300) or 100 µg (*n* = 595) of mRNA-1273.211, respectively (one enrolled participant was removed from all analyses because the participant received multiple COVID-19 vaccines outside of the study; Fig. 1). A historical control group (primary series) of 584 participants received two doses of 100-µg mRNA-1273 in the phase 3 COVE trial^1,2^. A second historical control group of 171 participants received one booster dose of 50-µg mRNA-1273 after two doses of 100-µg mRNA-1273 in a separate phase 2 study^15^. Participant demographic and baseline characteristics are shown in Table 1 for the two mRNA-1273.211 groups, for the primary series historical control group from the COVE trial and for the booster historical control group (mRNA-1273 booster dose, 50 µg)^1,2,15,21,22^. Demographics and baseline characteristics were overall similar in the 50-µg and 100-µg mRNA-1273.211 groups and the historical control groups. The mean age of the participants was 50.7 years (50-µg mRNA-1273.211), 53.0 years (100-µg mRNA-1273.211), 52.1 years (primary series historical control group) and 52.0 years (booster historical control group). In terms of gender, 56% were female in the 50-µg and 100-µg mRNA-1273.211 groups, 47% in the primary series historical control and 61% in the booster historical control group. Most participants were White (86% in 50-µg mRNA-1273.211, 87% in 100-µg mRNA-1273.211, 72% in the primary series historical control and 96% in the booster historical control groups), and 13%, 9%, 31% and 6% were Hispanic or Latinx in these groups, respectively. There was a higher percentage of participants who were Black or African American in the primary series historical control group (19%) compared to the groups that received 50 µg or 100 µg of mRNA-1273.211 (6%) or 50 µg of mRNA-1273 (3%). The percentages of participants with evidence of prior SARS-CoV-2 infection at baseline (day of the booster dose) were 1% (4/300) in the 50-µg mRNA-1273.211 group, 2% (13/595) in the 100-µg mRNA-1273.211 group and 4% (6/171) in the 50-µg mRNA-1273 group. The median (Q1, Q3) durations between the second dose of mRNA-1273 and the booster dose were 264 (246, 276) days (50-µg mRNA-1273.211), 294 (286, 303) days (100-µg mRNA-1273.211) and 219 (199, 231) days (50-µg mRNA-1273).

**Table 1:** Demographics and study participant characteristics

Characteristics *n* (%)*	50-µg mRNA-1273.211 booster dose	100-µg mRNA-1273.211 booster dose	Primary series historical control group; 100-µg mRNA-1273 primary series	Booster historical control group; 50-µg mRNA-1273 booster dose
	*n* = 300	*n* = 595	*n* = 584	*n* = 171
Age at screening (years)				
Mean (range)	50.7 (19, 85)	53.0 (19, 85)	52.1 (18–87)	52.0 (18, 87)
Age subgroup				
≥18 years and <65 years	238 (79)	449 (75)	438 (75)	133 (78)
≥65 years	62 (21)	146 (25)	146 (25)	38 (22)
Gender				
Male	133 (44)	264 (44)	308 (53)	67 (39)
Female	167 (56)	331 (56)	276 (47)	104 (61)
Ethnicity				
Hispanic or Latinx	38 (13)	52 (9)	183 (31)	10 (6)
Not Hispanic or Latinx	262 (87)	539 (91)	398 (68)	161 (94)
Not reported or unknown	0	4 (1)	3 (1)	0
Race				
White	257 (86)	520 (87)	419 (72)	164 (96)
Black or African American	19 (6)	34 (6)	109 (19)	5 (3)
Asian	9 (3)	18 (3)	13 (2)	1 (1)
American Indian or Alaska Native	1 (<1)	5 (1)	8 (1)	1 (1)
Native Hawaiian or Other Pacific Islander	0	1 (<1)	3 (1)	0
Multiracial	7 (2)	7 (1)	11 (2)	0
Other	4 (1)	6 (1)	16 (3)	0
Not reported or unknown	3 (1)	4 (1)	5 (1)	0
Body mass index (kg m^−^^2^)				
*n*	300	593	581	168
Mean (s.d.)	30.7 (7.6)	30.0 (7.1)	31.1 (7.9)	25.5 (3.2)
Duration between second injection of mRNA-1273 and the booster (days)				
*n*	300	595	NA	170
Median	264	294		219
Q1, Q3	246–276	286–303		199–231
Pre-booster RT–PCR SARS-CoV-2				
Negative	300 (100)	590 (99)	584 (100)	149 (100)
Positive	0	5 (1)	0	3 (2)
Missing	0	0	0	19 (98)
Pre-booster antibody to SARS-CoV-2 nucleocapsid^§^				
Negative	296 (99)	587 (99)	584 (100)	155 (100)
Positive	4 (1)	8 (1)	0	3 (2)
Missing	0	0	0	13 (98)
Pre-booster SARS-CoV-2 status^a^				
Negative	296 (99)	582 (98)	584 (100)	140 (82)
Positive	4 (1)	13 (2)	0	6 (4)
Missing	0	0	0	25 (15)

RT–PCR, reverse transcription polymerase chain reaction. *Percentages based on the number of participants in the safety set for study P205 or the total number of participants in the primary series historical control and booster historical control groups.

^a^Pre-booster (baseline). SARS-CoV-2 status was positive if there was evidence of prior COVID-19, defined as positive binding antibody against the SARS-CoV-2 nucleocapsid or positive RT–PCR at day 1. Negative SARS-CoV-2 status was defined as negative binding antibody against the SARS-CoV-2 nucleocapsid and a negative RT–PCR at day 1. ^§^Elecsys assay for binding antibody to SARS-CoV-2 nucleocapsid.

### Safety

**Fig. 2:**
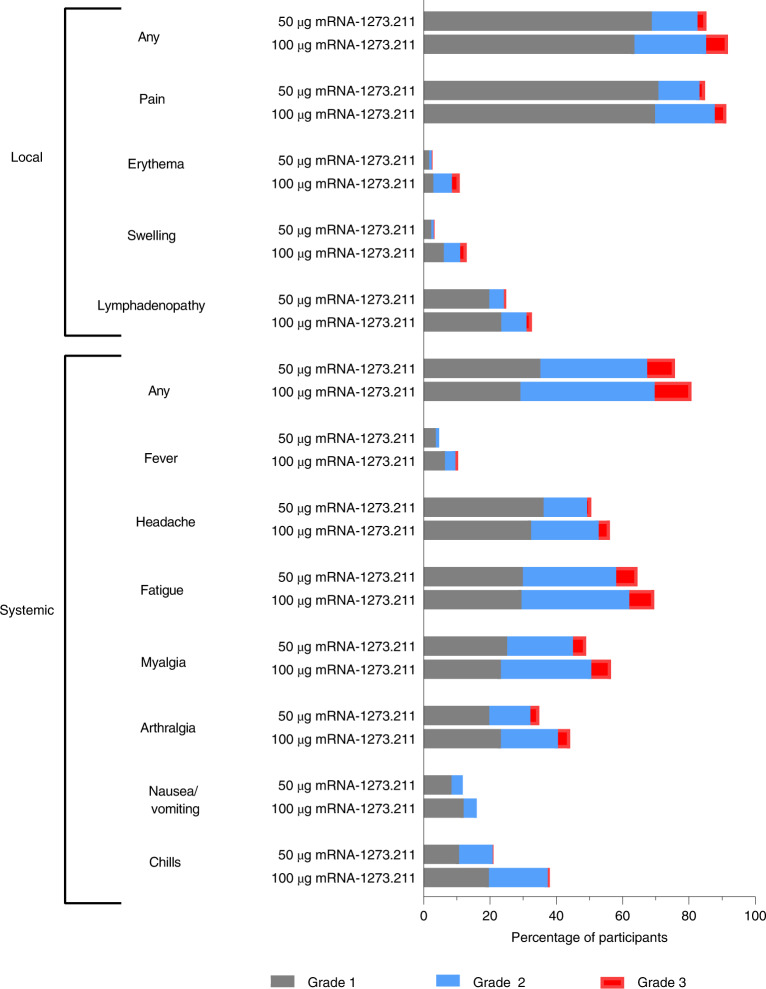
Solicited local and systemic adverse reactions. Percentages of participants who had a solicited local or systemic adverse reaction within 7 days after 50-µg and 100-µg doses of the mRNA-1273.211 booster.

The incidences of solicited local and systemic adverse reactions within 7 days after the mRNA-1273.211 booster injection (50-µg and 100-µg dose levels) are shown in Fig. 2, Supplementary Table 1 and Supplementary Fig. 3. The incidences of adverse reactions after the second dose of the mRNA-1273 primary series and after the 50-µg booster dose of the prototype mRNA-1273 were previously published^1,15,22^.

The most common solicited local adverse reactions within 7 days after administration of the 50-µg dose of mRNA-1273.211 was injection site pain (85%; 253/298). After administration of the 50-µg dose of mRNA-1273.211, the most common solicited systemic adverse reactions were fatigue (64%; 192/298), headache (51%; 151/298) and myalgia (49%; 146/298). After administration of the 100-µg dose of mRNA-1273.211, the most common solicited local adverse reaction was injection site pain (91%; 542/593), and the most common systemic adverse reactions were fatigue (70%; 413/593), headache (56%; 333/593) and myalgia (56%; 335/593). Most solicited adverse reactions in participants who received the 50-µg booster dose of mRNA-1273.211 were mild (local adverse reactions: grade 1 (69%; 205/298), grade 2 (14%; 41/298) and grade 3 (3%; 8/298); systemic adverse reactions: grade 1 (35%; 105/298), grade 2 (32%; 96/298) and grade 3 (8%; 25/298); Fig. 1 and Supplementary Table 1). Most solicited adverse reactions in participants who received the 100-µg mRNA-1273.211 booster dose were grade 1 or grade 2 (local adverse reactions: grade 1 (64%; 377/593), grade 2 (22%; 128/593) and grade 3 (7%; 39/593); systemic adverse reactions: grade 1 (29%; 173/593), grade 2 (40%; 240/593) and grade 3 (11%; 66/593); Fig. 1 and Supplementary Table 1). Overall, the 50-µg dose of mRNA-1273.211 had a similar reactogenicity profile to the 50-µg dose of the prototype mRNA-1273 and the second dose of the mRNA-1273 primary series, whereas the incidence of local and systemic adverse reactions was higher with the 100-µg dose of mRNA-1273.211 (refs. ^1,15,22^).

Unsolicited adverse events (AEs) regardless of the relationship to the vaccination up to 28 days after the 50-µg and 100-µg 1273.211 booster dose were reported in 21.0% (63/300) and 21.7% (129/595) of participants in these groups, respectively (Supplementary Table 2). No participants in the 50-µg group and 0.8% (5/595) of participants in the 100-µg group had serious adverse events (SAEs). These SAEs included hip pain, urinary tract infection, transient ischemic attack and bradycardia in a participant with history of cardiac disease; one of the five participants had multiple SAEs (cholelithiasis, urinary tract infection, urosepsis and nocturnal hypoxia). There were no fatal events; no SAEs related to vaccination, as assessed by the investigator; and no AEs leading to study discontinuations in either mRNA-1273.211 group up to 28 days after the booster dose. Overall, the frequency and types of unsolicited AEs were similar to those after the second dose of the mRNA-1273 primary series and the 50-µg mRNA-1273 booster dose^15,22^.

The median follow-up durations were 245 days and 210 days for the 50-µg and 100-µg mRNA-1273.211 booster groups, respectively. During this follow-up period, unsolicited AEs were reported in 63.0% (189/300) and 61.7% (367/ 595) of the participants in the 50-µg and 100-µg mRNA-1273.211 groups, respectively, regardless of the relationship to vaccination. Unsolicited AEs considered related to vaccination by the investigator were reported in 9.0% (27/300) of participants in the 50-µg group and 9.7% (58/595) of participants in the 100-µg group. No SAEs were considered by investigator to be related to vaccination during the follow-up period. There was one fatal event (cardiac arrest), which occurred in a 76-year-old male 159 days after the booster dose of mRNA-1273.211 (50 µg) and was not considered by the investigator to be related to vaccination. This participant had a history of obesity, hypertension, diabetes mellitus and dyslipidemia; was receiving cardiac medications; and was found unresponsive at home (an autopsy was not performed).

### Incidence of SARS-CoV-2 infections

Although the study was not designed to evaluate booster vaccine effectiveness, routine surveillance for SARS-CoV-2 infections with polymerase chain reaction (PCR) and anti-nucleocapsid antibody testing was performed at the clinic visits 28 days and 180 days after the mRNA-1273.211 booster doses. There were ten (10/296, 3.4%) and 70 (70/582, 12.0%) SARS-CoV-2 infections after the 50-µg and 100-µg mRNA-1273.211 booster doses, respectively. Routine surveillance at the day 181 clinic visits for some participants in the 100-µg mRNA-1273.211 group occurred during the Omicron wave in the United States, likely accounting for the higher frequency of infections in this group. From these SARS-CoV-2 infections, one (1/296, 0.3%) and 27 (27/582, 4.6%) in the 50-µg and 100-µg mRNA-1273.211 groups, respectively, were symptomatic and, hence, counted as COVID-19 events. There were no hospitalizations due to COVID-19. Lastly, there were 21 (21/171, 12.3%) participants with SARS-CoV-2 infections after the mRNA-1273 50-µg booster dose in the booster historical control group and 16 (16/149, 10.7%) participants with SARS-CoV-2 infections in the immunogenicity set (excluding participants with evidence of infection pre-booster) of the booster historical control group. Four of the 171 (2.3%) participants with a SARS-CoV-2 infection also had symptoms (COVID-19 events) in the booster historical control group.

### Immunogenicity

For the primary immunogenicity objective of assessing non-inferiority of the antibody response after the mRNA-1273.211 doses to that of the mRNA-1273 primary series (primary series historical control group), the geometric mean titers (GMTs) (analysis of covariance (ANCOVA) model) against the ancestral SARS-CoV-2 with the D614G mutation at 28 days after the 50-µg booster dose of mRNA-1273.211 (1,996.2 (1,777.9–2,241.4)) were higher than those at 28 days after the second 100-µg mRNA-1273 dose of the primary series in the historical control group (1,053.4 (967.2–1,147.2)) (Table 2) with a geometric mean ratio (GMR; 95% confidence interval (CI)) of 1.9 (1.7–2.2), meeting the non-inferiority criterion (GMR lower bound of 95% CI ≥0.67). The GMTs against the ancestral SARS-CoV-2 with the D614G mutation at 28 days after the 100-µg booster dose of mRNA-1273.211 (4,324.7 (3,974.6–4,705.6)) were higher than those at 28 days after the second 100-µg mRNA-1273 dose of the primary series in the primary series historical control group (1,087.3 (999.7–1,182.6)) with a GMR of 4.0 (3.6–4.4), also meeting the non-inferiority criterion (GMR lower bound of 95% CI ≥0.67) (Table 2). The seroresponse rate (SRR) against the ancestral SARS-CoV-2 with D614G at 28 days after the 50-µg booster dose of mRNA-1273.211 was 98.7% (96.6–99.6%) compared to 98.1% (96.7–99.1%) in the primary series historical control group, with a difference of 0.5% (−1.6% to 2.2%), which met the non-inferiority criterion (SRR difference lower bound of 95% CI > −10%) (Table 2). After the 100-µg booster dose of mRNA-1273.211, the SRR against the ancestral SARS-CoV-2 with D614G at 28 days was 99.8% (99.0–100.0%) compared to 98.1% (96.7–99.1%) in the primary series historical control group, with a difference of 1.7% (0.7–3.2%), which also met the non-inferiority criterion (SRR difference lower bound of 95% CI > −10%) (Table 2).

**Table 2:** Estimated neutralizing antibody titers against ancestral SARS-CoV-2 (D614G) and the Beta variant after the mRNA-1273 primary series and after the 50-µg and 100-µg booster dose of mRNA-1273.211

	Ancestral SARS-CoV-2 (D614G)		Beta (B.1.351)	Ancestral SARS-CoV-2 (D614G)
	50-µg, mRNA-1273.211 booster dose	Historical control, 100-µg mRNA-1273 primary series	50-µg, mRNA-1273.211 booster dose	Historical control, 100-µg mRNA-1273 primary series
	*n* = 299	*n* = 584	*n* = 299	*n* = 584
Pre-vaccination baseline, *n*†	299	584	297	584
GMT (95% CI)^§^	9.4 (9.1–9.7)	9.7 (9.3–10.1)	9.8 (NE–NE)	9.7 (9.3–10.1)
28 days after booster or 2nd dose, *n*	299	584	299	584
Estimated GMT (95% CI)¶	1,996.2 (1,777.9–2,241.4)	1,053.4 (967.2–1,147.2)	953.9 (844.1–1,078.0)	1,058.0 (966.9–1,157.7)
GMR (mRNA-1273.211-50-µg versus 100-µg mRNA-1273) (95% CI)	1.9 (1.7–2.2)		0.9 (0.8–1.0)	
SRRs n/N1 (%) (versus pre-vaccination)ǁ	295/299 (98.7)	573/584 (98.1)	291/297 (98.0)	573/584 (98.1)
(95% CI)‡	96.6–99.6	96.7–99.1	95.7–99.3	96.7–99.1
Difference % (95% CI)††	0.5 (−1.6 to 2.2)		−0.1 (−2.6 to 1.7)	

	100-µg, mRNA-1273.211 booster dose	Historical control, 100-µg mRNA-1273 primary series	100-µg, mRNA-1273.211 booster dose	Historical control, 100-µg mRNA-1273 primary series
	*n* = 578	*n* = 584	*n* = 584	*n* = 584
Pre-vaccination baseline n†	578	584	571	584
GMT (95% CI)^§^	9.4 (9.2–9.5)	9.7 (9.3–10.1)	9.8 (9.7–9.9)	9.7 (9.3–10.1)
28 days after booster or 2nd dose, *n*	578	584	578	584
Estimated GMT (95% CI)¶	4,324.7 (3,974.6–4,705.6)	1,087.3 (999.7–1,182.6)	1,574.6 (1,439.4–1,722.5)	1,085.7 (992.9–1,187.2)
GMR (mRNA-1273.211-100-µg versus 100-µg mRNA-1273) (95% CI)	4.0 (3.6–4.4)		1.5 (1.3–1.6)	
SRRs n/N1 (%) (versus pre-vaccination)ǁ	577/578 (99.8)	573/584 (98.1)	566/571 (99.1)	573/584 (98.1)
(95% CI)‡	(99.0–100.0)	(96.7–99.1)	(98.0–99.7)	(96.7–99.1)
Difference % (95% CI)††	1.7 (0.7–3.2)		1.0 (−0.4 to 2.6)	

NE, not estimated; ULOQ, upper limit of quantification.

Antibody values assessed by pseudovirus neutralizing antibody assay reported as below the LLOQ are replaced by 0.5× LLOQ, and values greater than ULOQ are replaced by the ULOQ if actual values are not available.

^*^Beta-specific antibody data are shown for the mRNA-1273.211 booster and for the ancestral SARS-Cov-2 with D614G for the primary series historical control group.

^†^Pre-vaccination baseline is before the first dose of mRNA-1273 in the primary series; *n* indicates the number of participants with non-missing data at the timepoint (baseline or post-baseline).

^§^95% CI is calculated based on the *t*-distribution of the log-transformed values for geometric mean value and then back-transformed to the original scale for presentation.

^¶^The log-transformed antibody levels were analyzed using an ANCOVA model with the treatment variable as fixed effect, adjusting for age group (<65 years and ≥65 years). The treatment variable corresponds to the primary series historical control group and each individual study group (mRNA-1273.211) dose levels. The resulting least squares means, differences of the least squares means and 95% CIs are back-transformed to the original scale for presentation.

^ǁ^Seroresponse at a participant level is defined as a change from below the LLOQ to equal to or above 4× LLOQ or at least a four-fold rise if baseline (titer before receiving the mRNA-1273 primary series) is equal to or above the LLOQ. Percentages are based on the number of participants with non-missing data at baseline and the corresponding timepoint (N1). For study participants with negative SARS-CoV-2 status before the primary series, antibody titers are imputed as <LLOQ at pre-dose 1 of the primary series. For participants without SARS-CoV-2 status information at pre-dose 1 of primary series, their pre-booster SARS-CoV-2 status was used to impute their SARS-CoV-2 status at pre-dose 1 of the primary series.

^‡^95% CI was calculated using the Clopper–Pearson method.

^††^95% CI was calculated using the Miettinen–Nurminen (score) confidence limits.

The Beta-specific GMTs at 28 days after the 50-µg booster dose of mRNA-1273.211 were 953.9 (844.1–1,078.0), and the GMTs against the ancestral SARS-CoV-2 with D614G at 28 days after the second dose of 100-µg mRNA-1273 of the primary series were 1,058.0 (966.9–1,157.7), with a GMR of 0.9 (0.8–1.0), meeting the non-inferiority criterion (Table 2). After the 100-µg booster dose of mRNA-1273.211, the Beta-specific GMTs at 28 days were 1,574.6 (1,439.4–1,722.5), and the GMTs against the ancestral SARS-CoV-2 with D614G 28 days after the second dose of 100-µg mRNA-1273 of the primary series were 1,085.7 (992.9–1,187.2), with a GMR of 1.5 (1.3–1.6), also meeting the non-inferiority criterion (Table 2). The SRR for the Beta variant at 28 days after the 50-µg booster dose of mRNA-1273.211 was 98.0% (95.7–99.3%), and the SRR against the ancestral SARS-CoV-2 with D614G was 98.1% (96.7–99.1%) in the primary series historical control group, with a difference of −0.1% (−2.6 to 1.7%), which met the non-inferiority criterion (Table 2). Lastly, the SRR against the Beta variant at 28 days after the 100-µg booster dose of mRNA-1273.211 was 99.1% (98.0–99.7%), and the SRR against the ancestral SARS-CoV-2 with D516G in the primary series historical control group was 98.1% (96.7–99.1%), with an SRR difference of 1.0% (−0.4 to 2.6%), which also met the non-inferiority criterion (Table 2).

For the immunogenicity objective of assessing non-inferiority and superiority of the antibody response after the mRNA-1273.211 booster dose (50-µg) to the mRNA-1273 booster (booster historical control group (50-µg mRNA-1273 booster)), the GMTs (mixed model for repeated measures (MMRM) model; Table 3) against the ancestral SARS-CoV-2 with the D614G mutation at 28 days after the 50-µg booster dose of mRNA-1273.211 (2,278.0 (2,074.0–2,502.1)) were higher than those at 28 days after the 50-µg booster dose of mRNA-1273 (1,782.7 (1,561.3– 2,035.6)), with a GMR (95% CI) of 1.28 (1.08–1.51). The GMTs against the ancestral SARS-CoV-2 with D614G mutation were also higher at 180 days after the 50-µg booster dose of mRNA-1273.211 (1,039.9 (926.4–1,167.3)) than that after the 50-µg mRNA-1273 dose (617.2 (525.1–725.5)), with a GMR of 1.68 (1.38–2.06). The superiority criterion was met (GMR lower bound of the 95% CI >1) at both timepoints, 28 days and 180 days after the booster dose, with a nominal alpha of 0.05.

**Table 3:** Neutralizing antibody estimated GMTs after the 50-µg mRNA-1273.211 and the 50-µg mRNA-1273 booster doses

Day after booster dose	mRNA-1273.211 50-µg booster dose GMT^a^ (95% CI)	mRNA-1273 50-µg booster dose GMT^a^ (95% CI)	GMR
Ancestral SARS-CoV-2 with D614G			
Day 29	2,278.0 (2,074.0–2,502.1)	1,782.7 (1,561.3–2,035.6)	**1.28 (1.08**–**1.51)**
Day 181	1,039.9 (926.4–1,167.3)	617.2 (525.1–725.5)	**1.68 (1.38**–**2.06)**
Beta			
Day 29	1,095.3 (981.1–1,222.7)	825.6 (706.6–964.7)	**1.33 (1.09**–**1.61)**
Day 181	343.5 (303.7–388.5)	125.2 (105.4–148.8)	**2.74 (2.22**–**3.40)**
Omicron			
Day 29	1,379.3 (1,209.9–1,572.4)	636.7 (529.1–766.3)	**2.17 (1.73**–**2.72)**
Day 181	308.0 (265.8–356.9)	133.0 (108.2–163.6)	**2.32 (1.80**–**2.98)**
Delta			
Day 29	1,483.0 (1,335.9–1,646.3)	838.8 (724.4–971.2)	**1.77 (1.48**–**2.12)**
Day 181	492.9 (438.3–554.2)	400.8 (340.4–471.8)	**1.23 (1.01**–**1.50)**

The number of participants was 282–295 for the mRNA-1273.211 group and 146–149 for the mRNA-1273 group.

^a^GMTs were estimated GLSM titers with an MMRM adjusting for age groups and pre-booster titer levels. The key measurements (bolded) of GMRs are for the estimated GMT after the 50-µg mRNA-1273.211 booster dose relative to the estimated GMT after the 50-µg mRNA-1273 booster dose. (Superiority).

The Beta-specific GMTs at 28 days after the 50-µg mRNA-1273.211 booster dose (1,095.3 (981.1–1,222.7)) were higher than those after the 50-µg mRNA-1273 booster dose (825.6 (706.6–964.7)), with a GMR of 1.33 (1.09–1.61) (Table 3). The Beta-specific GMTs were also higher 180 days after the 50-µg booster dose of mRNA-1273.211 (343.5 (303.7–388.5)) than those after the 50-µg mRNA-1273 dose (125.2 (105.4–148.8)), with a GMR of 2.74 (2.22–3.40). The superiority criterion was met (GMR lower bound of the 95% CI >1) at both timepoints, 28 days and 180 days after the booster dose, with a nominal alpha of 0.05.

The Omicron-specific GMTs 28 days after the 50-µg mRNA-1273.211 booster dose (1,379.3 (1,209.9–1572.4)) were higher than those 28 days after the 50-µg booster dose of mRNA-1273 (636.7 (529.1–766.3)), with GMR of 2.17 (1.73–2.72) (Table 3). At 180 days after the 50-µg booster dose of mRNA-1273.211, the Omicron-specific GMTs (308.0 (265.8–356.9)) were higher than those after the 50-µg mRNA-1273 booster dose (133.0 (108.2–163.6)), with GMR of 2.32 (1.80–2.98). The superiority criterion was met (GMR lower bound of the 95% CI >1) at both timepoints, 28 days and 180 days after the booster dose, with a nominal alpha of 0.05.

The Delta-specific GMTs 28 days after the 50-µg booster dose of mRNA-1273.211 (1,483.0 (1,335.9–1,646.3)) were higher than that after the 50-µg booster dose of mRNA-1273 (838.8 (724.4–971.2)), with a GMR of 1.77 (1.48–2.12) (Table 3). The Delta-specific GMTs at 180 days after the 50-µg mRNA-1273.211 booster dose were 492.9 (438.3–554.2) and were higher than those after the 50-µg booster dose of mRNA-1273 (400.8 (340.4–471.8)), with a GMR of 1.23 (1.01–1.50). The superiority criterion was met (GMR lower bound of the 95% CI >1) at both timepoints, 28 days and 180 days after the booster dose, with a nominal alpha of 0.05.

**Fig. 3:**
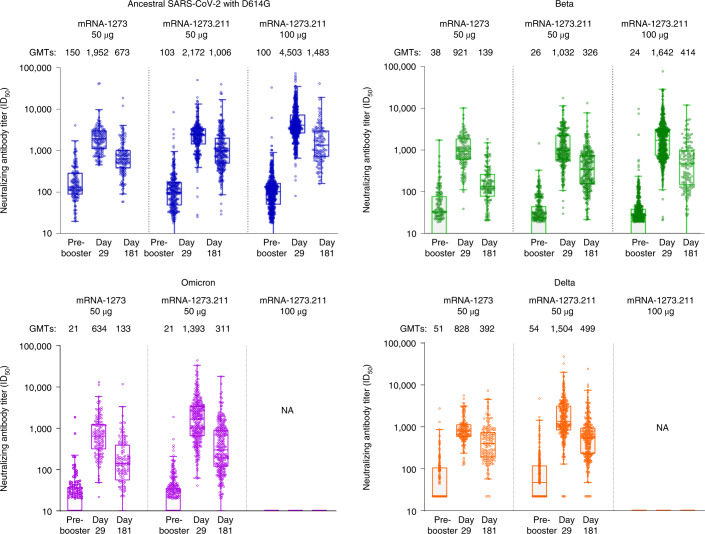
Observed neutralizing antibody GMTs against the ancestral SARS-CoV-2 and against the Beta, Omicron and Delta variants pre-booster, 28 days and 180 days after the mRNA-1273 and mRNA-1273.211 booster doses. The neutralizing antibody titers (ID_50_) in the pseudovirus assay against the ancestral SARS-CoV-2 with D614G (blue), the Beta variant (green), the Omicron variant (purple) and the Delta variant (orange) are shown for serum samples collected before the booster dose of 50 µg of mRNA-1273 (*n* = 146–149), 50 µg of mRNA-1273.211 (*n* = 286–299) or 100 µg of mRNA-1273.211 (*n* = 578 for pre-booster and day 29; *n* = 143 for day 181) (pre-booster), at 28 days after the booster dose (day 29) or at 180 days after the booster injection (day 181). The dots show the results from individual serum samples. The horizontal lines in the middle of the boxes show the median titers. The boxes extend from the 25th percentile to the 75th percentile. The whiskers show the maximum and minimum values within 1.5 times the IQR above the 75% and below the 25% percentiles. See Table 2 for the statistical comparison (MMRM model based) of the antibody response after the 50-µg mRNA-1273.211 booster dose to the antibody response after the 50-µg mRNA-1273 booster dose. (Non-inferiority). IQR, interquartile range (the difference between the 25th and 75th percentile); NA, not available.

The observed neutralizing antibody GMTs against the ancestral SARS-CoV-2 with D614G and Beta, after the mRNA-1273 primary series and after the mRNA-1273.211 booster doses, are shown in Supplementary Fig. 1. In addition, the observed neutralizing antibody GMTs after the 50-µg mRNA-1273 booster dose and after the mRNA-1273.211 booster doses against the ancestral SARS-CoV-2 with D614G, Beta, Delta and Omicron variants are shown in Fig. 3 (also see Supplementary Table 3 and Supplementary Fig. 2). The geometric mean fold rises (GMFRs) of the observed (not estimated) GMTs between pre-booster and day 29 post-booster indicate increased fold rises with the bivalent vaccine candidate, mRNA-1273.211 50-µg, compared to mRNA-1273 50-µg (Supplementary Table 4).

The observed GMTs (95% CIs) against the ancestral SARS-CoV-2 with D614G were 2,171.7 (1,952.3–2,415.7) at 29 days after the administration of the 50-µg booster dose of mRNA-1273.211 (*n* = 299); 1,951.7 (1,729.6–2,202.4) after the 50-µg dose of mRNA-1273 booster dose (*n* = 149); and 4,503.0 (4,165.0–4,868.5) after the 100-µg booster dose of mRNA-1273.211 (Fig. 3 and Supplementary Table 3). In addition, the GMTs against the ancestral SARS-CoV-2 with D614G were 1,006.1 (880.0–1,150.3) at 181 days after the 50-µg booster dose of mRNA-1273.211; 673.3 (578.2–784.0) after the 50-µg booster dose of mRNA-1273; and 1,482.6 (1,254.7–1,752.0) after the 100-µg booster dose of mRNA-1273.211 (Fig. 3 and Supplementary Table 3).

The observed Beta-specific GMTs (95% CIs) were 1,032.4 (912.8–1,167.8) at 29 days after the 50-µg dose of mRNA-1273.211; 920.5 (797.3–1,062.8) after the 50-µg dose of mRNA-1273; and 1,641.9 (1,504.0–1,792.6) after the 100-µg dose of mRNA-1273.211 (Fig. 3 and Supplementary Table 3). The Beta-specific GMTs were 326.4 (284.2–375.0) at 181 days after the 50-µg dose of mRNA-1273.211; 138.7 (115.6–166.5) after the 50-µg dose of mRNA-1273; and 413.8 (334.9–511.3) after the 100-µg mRNA-1273.211 booster (Fig. 3 and Supplementary Table 3).

The observed Omicron-specific GMTs (95% CIs) were 1,393.1 (1,196.9–1,621.5) at 29 days after the 50-µg dose of mRNA-1273.211 and 633.6 (531.7–754.9) after the 50-µg dose of mRNA-1273 (Fig. 3 and Supplementary Table 3). The Omicron-specific GMTs were 311.4 (264.2–367.1) at 181 days after the 50-µg dose of mRNA-1273.211 and 132.8 (105.9–166.6) after the 50-µg dose of mRNA-1273 (Fig. 3 and Supplementary Table 3).

The observed Delta-specific GMTs (95% CIs) were 1,504.0 (1,330.2–1,700.7) at 29 days after the 50-µg dose of mRNA-1273.211 and 827.8 (738.5–927.9) after the 50-µg dose of mRNA-1273 (Fig. 3 and Supplementary Table 3). The Delta-specific GMTs were 499.4 (438.6–568.6) at 181 days after the 50-µg dose of mRNA-1273.211 and 392.5 (333.1–462.4) after the 50-µg dose of mRNA-1273 (Fig. 3 and Supplementary Table 3).

The observed binding antibody titer (AU ml^−1^) for the ancestral SARS-CoV-2 with D614G was 592,403 (541,404–648,206) 28 days after the administration of the 50-µg booster dose of mRNA-1273.211; 480,711 (387,143–596,895) after the 50-µg dose of mRNA-1273 booster dose; and 803,379 (755,724–854,040) after the 100-µg booster dose of mRNA-1273.211 (Supplementary Fig. 4 and Supplementary Table 5). The Beta-specific binding antibody titers at 28 days after the administration of the 50-µg booster dose of mRNA-1273.211 were 299,241 (272,631–328,450); 199,067 (161,716–245,044) after the 50-µg dose of mRNA-1273; and 393,776 (369,877–419,220) after the 100-µg booster dose of mRNA-1273.211. The Gamma-specific binding antibody titers at 28 days after the administration of the 50-µg booster dose of mRNA-1273.211 were 313,177 (285,485–343,556); 231,094 (198,010–269,706) after the 50-µg dose of mRNA-1273; and 406,226 (381,449–432,613) after the 100-µg booster dose of mRNA-1273.211. The Alpha-specific binding antibody titers at 28 days after the administration of the 50-µg booster dose of mRNA-1273.211 were 454,210 (412,515–500,118); 368,165 (311,720–434,829) after the 50-µg dose of mRNA-1273; and 603,467 (566,823–642,479) after the 100-µg dose of mRNA-1273.211.

## Discussion

The results of this study indicate that the bivalent mRNA-1273.211 booster vaccine given to individuals who previously received a two-dose regimen of 100-µg of the mRNA-1273 primary series has a clinically acceptable safety and reactogenicity profile for both dose levels of mRNA-1273.211 (50 µg and 100 µg). The 50-µg mRNA-1273.211 booster dose has a safety and reactogenicity profile similar to that of the 50-µg booster dose of mRNA-1273 and to the second dose of the mRNA-1273 primary series^1,2,15^. The incidence of adverse reactions was higher with the 100-µg dose of mRNA-1273.211 compared to that after the 50-µg dose of mRNA-1273.211. This higher incidence of adverse reactions has also been observed with the prototype mRNA-1273 booster when administered at the 100-µg dose level^23^.

Neutralizing antibody (nAb) titers against SARS-CoV-2 variants remain detectable (GMT < 100) 7.0–9.8 months after immunization with the mRNA-1273 primary series at lower levels than the titers against the ancestral SARS-CoV-2 with D614G. Approximately 1 month after a first booster dose with mRNA-1273.211 (50 µg and 100 µg), nAbs rose to levels that exceeded the titers after immunization with the primary series (1.9-fold higher for ancestral SARS-CoV-2 with D614G and 7.1-fold higher for Beta with 50-µg dose of mRNA-1273.211), and the primary immunogenicity objective of non-inferiority to the primary series vaccination was met. Therefore, immunization with the primary series does not set a ceiling to the nAb response, and a booster dose of the bivalent vaccine elicits a robust response with titers that are likely to be protective against COVID-19 (refs. ^1,2,24,25^). The nAb titers after the 100-µg mRNA-1273.211 dose exceeded the titers after the 50-µg dose. However, the 50-µg mRNA-1273.211 booster dose titers were observed to be at least equivalent to those of the 50-µg dose of mRNA-1273, and the higher incidence of adverse reactions with the 100-µg booster dose of mRNA-1273 has previously led to the wide use of the 50-µg booster dose of mRNA-1273 in adults^23,26^.

A first booster dose with the the previously authorized 50 ug mRNA-1273 booster elicited a potent neutralizing response against the ancestral SARS-CoV-2 with D614G and the Beta, Delta and Omicron BA.1 variants^15^. However, the antibody titers, especially for Beta and Omicron, waned 6 months after the booster dose^10^. To evaluate whether a bivalent booster can enhance the breadth and the durability of the nAb response, we compared these responses with the mRNA-1273.211 booster (50 µg). The nAb titers against ancestral SARS-CoV-2 and all three variants significantly increased 1 month and 6 months after boost with mRNA-1273.211 compared to mRNA-1273, and the superiority immunogenicity objective was met. Given that 3.4% and 10.7% of the mRNA-1273.211 (50 µg) and mRNA-1273 booster (50 µg) vaccinees, respectively, had evidence of a SARS-CoV-2 infection, it is unlikely that natural infection led to increased antibody titers in the mRNA-1273.211 group (50 µg) or influenced the booster-to-booster comparison in favor of the mRNA-1273.211 group. In addition, the binding antibody responses (ancestral SARS-CoV-2, Beta, Alpha and Gamma) were consistently higher with the bivalent booster vaccine than the mRNA-1273 booster at approximately 1 month after the booster dose. Although it is conceivable that the presentation of multiple antigens after the bivalent booster vaccine induces further maturation and evolution of the humoral response, evaluation of antigen-reactive B cells in the B cell compartment is needed to further elucidate the mechanisms of enhancing the immune response^27–29^.

This study had several limitations. It was not designed and randomized to compare different booster candidates or dose levels head to head, and the evaluation of booster candidates was sequential and open-label. Neutralization results from different groups were not generated in the laboratory at the same time, and the study was not designed to evaluate vaccine effectiveness or multiple intervals between the primary immunization series and the booster dose. We adjusted for the pre-booster titer levels when comparing the antibody responses between mRNA-1273.211 and mRNA-1273 booster groups to help address this limitation.

Overall, the bivalent mRNA-1273.211 booster vaccine had a clinically acceptable safety profile, similar to mRNA-1273 when administered at the 50-µg dose level. In addition, the mRNA-1273.211 vaccine (50 µg) elicited robust and persistent antibody responses against multiple variants of concern, even when some of these variants were not contained in the vaccine. Cross-neutralization of multiple variants and the potency and durability of the antibody response appear to be advantages of bivalent booster vaccines that contain both the ancestral SARS-CoV-2 and variant spike sequences, and such vaccines may represent an important strategy as we continue to respond to emerging SARS-CoV-2 variants.

## Methods

This is part A of an open-label, non-randomized phase 2/3 ongoing study (NCT04927065) to evaluate the safety, reactogenicity and immunogenicity of a single booster dose of the bivalent mRNA candidate vaccine mRNA-1273.211 (see the ‘Trial vaccine’ section below) in adults who had previously received the primary series of two doses of 100-µg mRNA-1273 in the phase 3 COVE trial at least 6 months earlier (Fig. 1)^1,2^. Study participants are being followed for approximately 12 months after administration of the booster dose. Amendments to the protocol are summarized in the Supplementary Information.

Participants (*n* = 896) ≥18 years of age who were compliant (not withdrawn or discontinued early) in the phase 3 COVE trial and who had completed a two-dose primary series of mRNA-1273 at least 6 months prior were enrolled across nine clinical sites in the United States from 28 May 2021 to 15 July 2021 (NCT04927065). A total of 300 participants were enrolled in the 50-µg mRNA-1273.211 booster group and 596 participants in the 100-µg mRNA-1273.211 booster group (first booster doses; Fig. 1). Participants who had a history of SARS-CoV-2 infection in the COVE trial or who were found to have evidence of SARS-CoV-2 infection at study screening were excluded (see inclusion and exclusion criteria in the supplement and online protocol). Screening for SARS-CoV-2 infections before the booster dose was based on SARS-CoV-2 PCR as well as SARS-CoV-2 nucleocapsid antibody testing. Because nucleocapsid antibody testing results can take several days, four of 300 (1.3%) participants in the 50-µg mRNA-1273.211 group who had a positive antibody test result at baseline had already received the booster dose.

In addition, 584 participants who received the two-dose primary series of mRNA-1273 (100 µg) were randomly selected from the random sub-cohort for Immunogenicity of the phase 3 COVE trial^1,2,22^. This COVE group serves as a historical control group when comparing the antibody responses after the mRNA-1273.211 booster doses (50 µg and 100 µg) with that after the mRNA-1273 series (see the ‘Statistical analysis’ section below).

Lastly, 171 participants ≥18 years of age enrolled in a separate phase 2 study (NCT04405076) and received the authorized mRNA-1273 booster (50 µg, first booster dose) at least 6 months after completing the mRNA-1273 primary series, and they serve as a historical control group when comparing the antibody responses between the mRNA-1273.211 (50 µg) and mRNA-1273 (50 µg) boosters (Fig. 1)^15^.

The trials have been conducted in accordance with the International Council for Technical Requirements for Registration of Pharmaceuticals for Human Use, Good Clinical Practice Guidance and applicable government regulations. The central institutional review board (Advarra) approved the protocol and consent forms. All participants provided written informed consent. The clinical trial was submitted to ClinicalTrials.gov within 20 days of initiation and had no independent safety monitoring board given that it was an open-label study and the sponsor monitored all reported AEs. Participants were compensated for time and travel.

### Trial vaccine

The mRNA-1273 vaccine contains an mRNA that encodes the spike glycoprotein of the Wuhan-Hu-1 isolate of SARS-CoV-2 encapsulated in a lipid nanoparticle (LNP). The booster doses of mRNA-1273 were administered at a dose level of 50-µg mRNA-1273 in a 0.5-ml volume. The bivalent mRNA-1273.211 vaccine uses the same LNP containing equal amounts of mRNA that encode for the spike glycoproteins of Wuhan-Hu-1 and the Beta (B.1.351) variant. The booster doses of mRNA-1273.211 were administered at dose levels of 50 µg or 100 µg.

### Study outcomes

The primary safety objective was to evaluate the safety and reactogenicity of a 50-µg or 100-µg mRNA‑1273.211 administered as a single booster dose. Reactogenicity included solicited local and systemic adverse reactions that occurred ≤7 days after the booster injection as recorded daily by participants. Unsolicited AEs were recorded by study sites for 28 days after booster administration. SAEs, medically attended adverse events (MAAEs) and adverse events of special interest (AESIs) were recorded by the study sites for the entire study period (~12 months).

There were two pre-specified immunogenicity objectives in the study. The primary objective was to demonstrate a non-inferior antibody response against the ancestral SARS-CoV-2 and the Beta variant 28 days after the booster dose of 50-µg or 100-µg mRNA‑1273.211 candidate vaccine compared to the antibody response 28 days after the second dose of the 100-µg mRNA‑1273 primary series in the historical control group based on endpoints of the antibody GMR and group difference in SRRs (see the ‘Statistical analysis’ section below).

The second pre-specified immunogenicity objective was to perform non-inferiority and superiority comparisons of the antibody response of the 50-µg mRNA-1273.211 booster candidate with the antibody response of the 50-µg mRNA-1273 booster dose for the ancestral SARS-CoV-2 and for the Beta, Delta and Omicron variants based on the endpoint of the antibody GMR 28 days and 180 days after the booster doses (see the ‘Statistical analysis’ section below). Comparison of the 100-µg mRNA‑1273.211 booster vaccine candidate to the 50-µg mRNA-1273 booster dose was not part of the immunogenicity objective to compare the two booster vaccines, and, therefore, antibody titers against Omicron and Delta after the 100-µg mRNA‑1273.211 were not evaluated.

### Immunogenicity assays

nAb GMTs at 50% inhibitory dilutions (ID_50_) were assessed in validated SARS-CoV-2 spike pseudotyped lentivirus neutralization assays (PsVNAs). Titers were generated against pseudoviruses containing the SARS-CoV-2 full-length spike proteins for the ancestral virus with the D614G mutation and the Beta, Delta and Omicron (BA.1) variants. GMTs were also assessed in an anti-spike protein-binding IgG antibody assay (Meso Scale Discovery (MSD)) against the ancestral SARS-CoV-2 with D614G, Gamma (P.1), Alpha (B.1.1.7) and Beta variants. Immunogenicity assays are further described in the supplement.

### Statistical analysis

Safety was evaluated in the safety set with participants who received the 50-µg or 100-µg mRNA-1273.211 booster doses, and solicited adverse reactions were assessed in the solicited safety set (see further information on analysis sets in Supplementary Information). The numbers and percentages of participants with any solicited local and systemic adverse reactions occurring within 7 days after boost are provided. Unsolicited AEs, SAEs, severe AEs, MAAEs, AESIs and AEs leading to study discontinuation are also summarized.

The primary immunogenicity objective for 50-µg and 100-µg mRNA-1273.211 booster doses was assessed in the immunogenicity set consisting of all participants who received the booster dose and had antibody data available at the pre-booster and the day 29 visit with no major protocol deviations. The primary immunogenicity objectives were (1) to demonstrate a non-inferior antibody response 28 days after the booster dose against the ancestral SARS-CoV-2 (D614G) compared to the antibody response after the second dose of the mRNA-1273 primary series (historical control group) against the ancestral SARS-CoV-2 (D614G) and (2) to demonstrate a non-inferior antibody response 28 days after the booster against the Beta variant compared to the antibody response after the second dose of the mRNA-1273 primary series (historical control group) against the ancestral SARS-CoV-2 (D614G) The clinical endpoints assessed to demonstrate non-inferiority included the GMR and the group difference in SRRs (Supplementary Methods). Non-inferiority was considered met if the lower bound of the 95% CI (two-sided) of the GMR was ≥0.67 (1/1.5, non-inferiority margin of 1.5). Non-inferiority of the difference in SRR was considered met if the lower bound of the 95% CI (two-sided) of the SRR difference was > −10%. Seroresponse was defined as a four-fold titer rise for participants with a pre-vaccination baseline titer (titer before receiving the mRNA-1273 primary series), ≥lower limit of quantification (LLOQ) of the neutralizing antibody assay or ≥4 times the LLOQ of the assay for those participants with a pre-vaccination baseline <LLOQ.

For the immunogenicity objective of the booster-to-booster comparison, the antibody responses elicited by the 50-µg mRNA-1273.211 booster dose were compared to those elicited by the 50-µg mRNA-1273 booster dose (booster historical control group (50-µg mRNA-1273 booster)) at 28 days and 180 days after boost. This objective was pre-specified in a separate analysis plan (outside the study protocol). The booster-to-booster comparison was assessed using the immunogenicity set (excluding participants with evidence of infection before booster). The clinical endpoint assessed to demonstrate non-inferiority and superiority was based on GMR; non-inferiority (antibody response of mRNA-1273.211 50-µg over mRNA-1273 50-µg) was considered met if the lower bound of GMR 95% CI was ≥0.67, and superiority was considered met if the lower bound of 95% CI was >1. All tests were performed at a nominal alpha level of 0.05 (two-sided).

For the immunogenicity endpoints, nAb titers were analyzed using an ANCOVA model for the antibody response non-inferiority assessment compared to the mRNA-1273 primary series to adjust for age groups. Specifically, the model included log-transformed antibody titers 28 days after boost and 28 days after second dose of the primary series as the dependent variables, treatment groups (50-µg mRNA-1273.211 booster dose versus 100-µg primary series or 100-µg mRNA-1273.211 booster dose versus 100-µg primary series) as explanatory variables and adjustment for age groups (<65 years and ≥65 years). For the booster-to-booster non-inferiority and superiority comparisons, an MMRM (given multiple timepoints: day 29 and day 181 after boost) was used. The model included treatment groups, clinic visits, treatment by visit interaction (as the immunization effect can vary with time) and adjustment for age groups and pre-booster titer levels. An unstructured covariance structure was used to model the within-participant covariance. In addition, the 95% CIs (two-sided) of seroresponse differences between groups were based on the Miettinen–Nurminen (score) method (see supplement for additional information).

The target enrollment of 50-µg mRNA-1273.211 was approximately 300 participants, and 270 participants were assumed to be evaluable. Additionally, 526 participants were also assumed to be evaluable in the primary series historical control group. With this sample size, there is approximately 90% power to reject all null hypotheses for the primary immunogenicity objectives based on the GMR and SRR difference endpoints against the ancestral SARS-CoV-2 and the Beta variant (at a two-sided alpha of 5.0%) with the following underlying assumptions: the true GMRs (50-μg mRNA-1273.211 booster versus 100-μg mRNA-1273 primary series) against the ancestral SARS-CoV-2 and Beta are 1, and the standard deviation of the log-transformed titer is 1.5, with a non-inferiority margin of 1.5; the true SRRs against the ancestral SARS-CoV-2 and Beta after the booster dose of mRNA-1273.211 50-μg is 90%, and the SRR against ancestral SARS-CoV-2 after mRNA-1273 primary series is also 90%, and the non-inferiority margin for the SRR difference is 10%.

All analyses were conducted using SAS version 9.4 or higher.

### Study eligibility criteria

#### Inclusion criteria

Each participant must meet all of the following criteria to be enrolled in this study:

- Male or female, at least 18 years of age at the time of consent (screening visit).
- Investigator’s assessment that participant understands and is willing and physically able to comply with protocol-mandated follow-up, including all procedures.
- Participant has provided written informed consent for participation in this study, including all evaluations and procedures as specified in this protocol.
- Female participants of non-childbearing potential may be enrolled in the study.
- Non-childbearing potential is defined as surgically sterile (history of bilateral tubal ligation, bilateral oophorectomy or hysterectomy) or postmenopausal (defined as amenorrhea for ≥12 consecutive months before screening (day 0) without an alternative medical cause). A follicle-stimulating hormone level may be measured at the discretion of the investigator to confirm postmenopausal status.
- Female participants of childbearing potential may be enrolled in the study if the participant fulfills all of the following criteria:
- Has a negative pregnancy test on the day of vaccination (day 1).
- Has practiced adequate contraception or has abstained from all activities that could result in pregnancy for at least 28 days before day 1.
- Has agreed to continue adequate contraception through 3 months after vaccination.
- Is not currently breastfeeding.
- Adequate female contraception is defined as consistent and correct use of a US Food & Drug Administration-approved contraceptive method in accordance with the product label.
- Participant must have been previously enrolled in the mRNA-1273 COVE study, must have received two doses of mRNA-1273 in part A of that study (that is, is already unblinded and aware of their actual treatment), with their second dose at least 6 months before enrollment in this mRNA-1273 study, and must be currently enrolled and compliant in that study (that is, has not withdrawn or discontinued early).

#### Exclusion criteria

Participants meeting any of the following criteria at the screening visit, unless noted otherwise, will be excluded from the study:

- Had significant exposure to someone with SARS-CoV-2 infection or COVID-19 in the past 14 days, as defined by the US Centers for Disease Control and Prevention as a close contact of someone who has COVID-19.
- Has known history of SARS-CoV-2 infection, including during the mRNA-1273 COVE study.
- Is acutely ill or febrile (temperature ≥38.0 °C (100.4 °F)) fewer than 72 hours before or at the screening visit or day 1. Participants meeting this criterion may be rescheduled and will retain their initially assigned participant number.
- Currently has symptomatic acute or unstable chronic disease requiring medical or surgical care, to include significant change in therapy or hospitalization for worsening disease, at the discretion of the investigator.
- Has a medical, psychiatric or occupational condition that may pose additional risk as a result of participation or that could interfere with safety assessments or interpretation of results, according to the investigator’s judgment.
- Has a current or previous diagnosis of immunocompromising condition, to include HIV, immune-mediated disease requiring immunosuppressive treatment or other immunosuppressive condition.
- Has received systemic immunosuppressants or immune-modifying drugs for >14 days in total within 6 months before screening (for corticosteroids, ≥10 mg per day of prednisone equivalent) or is anticipating the need for immunosuppressive treatment at any time during participation in the study.
- Has known or suspected allergy or history of anaphylaxis, urticaria or other significant adverse reaction to the vaccine or its excipients.
- Has a medical history consistent with an AESI (as described in the Appendix).
- Coagulopathy or bleeding disorder considered a contraindication to intramuscular injection or phlebotomy.
- Has received or plans to receive any licensed vaccine ≤28 days before the injection (day 1) or a licensed vaccine within 28 days before or after the study injection, with the exception of influenza vaccines, which may be given 14 days before or after receipt of a study vaccine.
- Has received systemic immunoglobulins or blood products within 3 months before the screening visit (day 0) or plans for receipt during the study.
- Has donated ≥450 ml of blood products within 28 days before the screening visit or plans to donate blood products during the study.
- Plans to participate in an interventional clinical trial of an investigational vaccine or drug while participating in this study.
- Is an immediate family member or household member of study personnel, study site staff or sponsor personnel.
- Is currently experiencing an SAE in the mRNA-1273 COVE study at the time of screening for this study.

### Immunogenicity assays

#### SARS-CoV-2 spike PsVNA

SARS-CoV-2 nAbs in samples were assessed using the validated SARS-CoV-2 spike PsVNA in 293T/ACE2 cells. The PsVNA quantifies nAbs using lentivirus particles that express SARS-CoV-2 Wuhan-Hu-1 full-length spike proteins with the following amino acid substitutions (prototype (D614G); Beta (B.1.351 (501Y-V2); L18F, D80A, D215G, Δ242-244, R246I, K417N, E484K, N501Y, D614G and A701V); Omicron [B.1.1.529; BA.1] with the following amino acid changes in the spike protein [A67V, ∆H69-V70, T95I, G142D, ∆143-145, ∆211/L212II, ins214EPE, G339D, S371L, S373P, S375F, K417N, N440K, G446S, S477N, T478K, E484A, Q493R, G496S, Q498R, N501Y, Y505H, T547K, D614G, H655Y, N679K, P681H, N764K, D796Y, N856K, Q954H, N969K, and L981F]; and Delta ((B.1.617.2; AY.3); T19R, G142D, Δ156- 157, R158G, L452R, T478K, D614G, P681R and D950N)) on their surface and contain a firefly luciferase reporter gene for quantitative measurements of infection in transduced 293T cells expressing high levels of ACE2 (293T/ACE2 cells) by relative luminescence units (RLU). Serial dilution of antibodies was used to produce a dose–response curve. Neutralization was measured as the serum dilution at which RLU was reduced by 50% (ID_50_) relative to mean RLU in virus control wells (cells + virus but no sample) after subtraction of mean RLU in cell control wells (cells only). Positive controls were included on each assay plate to follow stability over time.

#### SARS-CoV-2 MSD assay

The validated MSD assay (SARSCOV2S2P (VAC83); https://www.mesoscale.com/products/sars-cov-2-panel-2-igg-k15383u/) uses an indirect, quantitative, electrochemiluminescence method to detect SARS-CoV-2-binding IgG antibodies to the SARS-CoV-2 full-length spike protein (Wuhan-Hu-1 ancestral SARS-CoV-2; Beta (B.1.351; 501Y-V2) with the following amino acid changes in the spike protein (L18F, D80A, D215G, Δ242-244, R246I, K417N, E484K, N501Y, D614G and A701V); Alpha (B.1.1.7; V1) with the following amino acid changes in the spike protein (ΔH69-V70, ΔY144Y, N501Y, A570D, D614G, P681H, T761I, S982A and D1118H); and Gamma (P.1; V3) with the following amino acid changes in the spike protein (L18F, T20N, P26S, D138Y, R190S, K417T, E484K, N501Y, D614G, H655Y, T1027I and V1176F)) in human serum. The assay was performed by PPD. The assay is based on MSD technology, which employs capture molecule MULTI-SPOT microtiter plates fitted with a series of electrodes.

### Pre-specified immunogenicity endpoints

There were four pre-specified immunogenicity endpoints for the primary immunogenicity objective:

1. Two non-inferiority endpoints based on the day 29 post-boost GMR (GMT against ancestral SARS-CoV-2 after boost/GMT against ancestral SARS-CoV-2 after the second dose of the primary series and GMT against the Beta variant/GMT against the ancestral SARS-CoV-2 after the second dose of the primary series, with a non-inferiority margin of 1.5). The GMR-based endpoint was considered met if the lower bound of the 95% CI of each GMR was ≥0.67 (1/1.5).
2. Two non-inferiority endpoints based on the day 29 post-boost difference in the SRR (SRR difference between the SRR against the ancestral SARS-CoV-2 after boost and the SRR against ancestral SARS-CoV-2 after the second dose of the primary series; and SRR against the Beta variant after boost and the SRR against ancestral SARS-CoV-2 after the second dose of the primary series). The SRR difference-based endpoint was considered met if the lower bound of the 95% CI was > −10%.

An ANCOVA model was used to assess non-inferiority for the primary immunogenicity objectives. The model included log-transformed antibody titers day 29 after boost and 28 days after the second dose of the primary series as the dependent variables, treatment groups (50-µg mRNA-1273.211 booster dose versus 100-µg primary series or 100-µg mRNA-1273.211 booster dose versus 100-µg primary series) as explanatory variables and adjustment for age groups (<65 years and ≥65 years). The geometric least squares mean (GLSM) and corresponding two-sided 95% CI for the antibody titers for each treatment group were calculated. The GLSM and the corresponding 95% CI results in log-transformed scale estimated from the model were back-transformed to obtain estimates in the original scale. GMR, estimated by the ratio of GLSM and the corresponding two-sided 95% CI, was used to assess the treatment difference.

To assess non-inferiority of the antibody response based on SRR, the number and percentage (rate) of participants achieving seroresponse 28 days after the booster dose or after the second dose in the historical control group were summarized, with 95% CI calculated using the Clopper–Pearson method for each group. The difference of SRRs between 50-µg or 100-µg mRNA-1273.211 28 days after the booster dose and 100-µg mRNA-1273 primary 28 days after the second dose were calculated with 95% CI using the Miettinen–Nurminen (score) method.

For the primary immunogenicity endpoints, a sample size of 300 participants in the 50-µg mRNA-1273.211 group and 584 participants in the 100-µg mRNA-1273.211 group corresponded to at least 75% power for the hypothesis testing in each group.

For the pre-specified booster-to-booster comparisons (antibody responses after the 50-µg mRNA-1273.211 versus 50-µg mRNA-1273 at 28 days and 180 days after the booster doses), the MMRM was used to analyze the post-boost observations. For the antibody response against the ancestral SARS-CoV-2 and variants (Beta, Omicron and Delta), the model included treatment groups, clinic visits, treatment by visit interaction and adjustment for age groups and pre-booster titer levels. An unstructured covariance structure was used to model the within-participant covariance. The GLSM and the corresponding 95% CI for each treatment group were estimated from the model at each post-boost timepoint. The GMR (GLSM titer after the mRNA-1273.211 booster dose over the mRNA-1273 booster dose) was estimated from the model, and the corresponding 95% CI was provided for treatment comparisons at each timepoint.

### Reporting summary

Further information on research design is available in the Nature Research Reporting Summary linked to this article.

## Online content

Any methods, additional references, Nature Research reporting summaries, source data, extended data, supplementary information, acknowledgements, peer review information; details of author contributions and competing interests; and statements of data and code availability are available at 10.1038/s41591-022-02031-7.

## Supplementary information

Supplementary Information List of Investigators, Supplementary Tables 1–5, Supplementary Figs. 1–4 and Protocol Amendment Summary of Changes

Reporting Summary

## Data Availability

As the trial is ongoing, access to patient-level data and supporting clinical documents with qualified external researchers may be available upon request and subject to review once the trial is complete. Such requests can be made to Moderna, Inc., 200 Technology Square, Cambridge, MA 02139. A materials transfer and/or data access agreement with the sponsor will be required for accessing of shared data.
