# Supplementary material for: Safety, immunogenicity and antibody persistence of a bivalent Beta-containing booster vaccine against COVID-19: a phase 2/3 trial
Source: Nat Med. 2022 Oct 6;28(11):2388–97. doi: 10.1038/s41591-022-02031-7 (PMC9671805; doi:10.1038/s41591-022-02031-7)
Supplement: Supplementary file 2 — Reporting Summary [file 41591_2022_2031_MOESM2_ESM.pdf]

nature portfolio

Corresponding author(s): Spyros Chalkias, M.M.

Last updated by author(s): August 10, 2022

Reporting Summary

Nature Portfolio wishes to improve the reproducibility of the work that we publish. This form provides structure for consistency and transparency in reporting. For further information on Nature Portfolio policies, see our Editorial Policies and the Editorial Policy Checklist.

Statistics

For all statistical analyses, confirm that the following items are present in the figure legend, table legend, main text, or Methods section.

n/a	Confirmed	
☐	☒	The exact sample size (n) for each experimental group/condition, given as a discrete number and unit of measurement
☐	☒	A statement on whether measurements were taken from distinct samples or whether the same sample was measured repeatedly
☐	☒	The statistical test(s) used AND whether they are one- or two-sided<br>*Only common tests should be described solely by name; describe more complex techniques in the Methods section.*
☒	☐	A description of all covariates tested
☒	☐	A description of any assumptions or corrections, such as tests of normality and adjustment for multiple comparisons
☐	☒	A full description of the statistical parameters including central tendency (e.g. means) or other basic estimates (e.g. regression coefficient) AND variation (e.g. standard deviation) or associated estimates of uncertainty (e.g. confidence intervals)
☐	☒	For null hypothesis testing, the test statistic (e.g. F, t, r) with confidence intervals, effect sizes, degrees of freedom and P value noted<br>*Give P values as exact values whenever suitable.*
☒	☐	For Bayesian analysis, information on the choice of priors and Markov chain Monte Carlo settings
☒	☐	For hierarchical and complex designs, identification of the appropriate level for tests and full reporting of outcomes
☒	☐	Estimates of effect sizes (e.g. Cohen's d, Pearson's r), indicating how they were calculated

Our web collection on statistics for biologists contains articles on many of the points above.

Software and code

Policy information about availability of computer code

Data collection	*Provide a description of all commercial, open source and custom code used to collect the data in this study, specifying the version used OR state that no software was used.*
Data analysis	All analyses were conducted using SAS Version 9.4 or higher.

For manuscripts utilizing custom algorithms or software that are central to the research but not yet described in published literature, software must be made available to editors and reviewers. We strongly encourage code deposition in a community repository (e.g. GitHub). See the Nature Portfolio guidelines for submitting code & software for further information.

Data

Policy information about availability of data

All manuscripts must include a data availability statement. This statement should provide the following information, where applicable:

- Accession codes, unique identifiers, or web links for publicly available datasets
- A description of any restrictions on data availability
- For clinical datasets or third party data, please ensure that the statement adheres to our policy

As the trial is ongoing, access to patient-level data and supporting clinical documents with qualified external researchers may be available upon request and subject to review once the trial is complete. Such requests can be made to Moderna, Inc., 200 Technology Square, Cambridge, MA 02139.

Field-specific reporting

Please select the one below that is the best fit for your research. If you are not sure, read the appropriate sections before making your selection.

☒ Life sciences ☐ Behavioural & social sciences ☐ Ecological, evolutionary & environmental sciences

For a reference copy of the document with all sections, see nature.com/documents/nr-reporting-summary-flat.pdf

Life sciences study design

All studies must disclose on these points even when the disclosure is negative.

Sample size	The target enrollment of 50 µg mRNA-1273.211 was approximately 300 participants and 270 participants were assumed to be evaluable. Additionally, 526 participants were also assumed to be evaluable in the primary series historical control group. With this sample size there is approximately 90% power to reject all null hypotheses for the primary immunogenicity objectives based on the GMR and SRR-difference endpoints against the ancestral SARS-CoV-2 and the Beta variant (at 2-sided alpha of 5.0%) with the following underlying assumptions: the true GMRs (50 µg mRNA-1273.211 booster vs. 100 µg mRNA-1273 primary series) against the ancestral SARS-CoV-2 and Beta are 1 and the standard deviation of the log transformed titer is 1.5, with a non-inferiority margin of 1.5; the true SRRs against the ancestral SARS-CoV-2 and Beta after the booster dose of mRNA-1273.211 50 µg is 90%, and the SRR against ancestral SARS-CoV-2 after mRNA-1273 primary series is also 90%, and the non-inferiority margin for the SRR difference is 10%.
Data exclusions	No data were excluded from the analyses.
Replication	This was a clinical study which collected one sample at each time point.
Randomization	This was not a randomized trial. Demographics and baseline characteristics were overall comparable in the 50 and 100-µg mRNA-1273.211 groups and the historical control groups.
Blinding	This was an open-label trial.

Reporting for specific materials, systems and methods

We require information from authors about some types of materials, experimental systems and methods used in many studies. Here, indicate whether each material, system or method listed is relevant to your study. If you are not sure if a list item applies to your research, read the appropriate section before selecting a response.

Materials & experimental systems

n/a	Involved in the study	
☒	☐	Antibodies
☒	☐	Eukaryotic cell lines
☒	☐	Palaeontology and archaeology
☒	☐	Animals and other organisms
☐	☒	Human research participants
☐	☒	Clinical data
☒	☐	Dual use research of concern

Methods

n/a	Involved in the study	
☒	☐	ChIP-seq
☒	☐	Flow cytometry
☒	☐	MRI-based neuroimaging

Human research participants

Policy information about studies involving human research participants

Population characteristics	Please see Table 1 in the manuscript. Demographics and baseline characteristics were overall comparable in the 50 and 100-µg mRNA-1273.211 groups and the historical control groups. . The mean age of the participants was 50.7 years (50 µg mRNA-1273.211), 53.0 years (100 µg mRNA-1273.211), 52.1 years (primary series historical control group) and 52.0 years (booster historical control group). In terms of gender, 56% were female in the 50 and 100 µg mRNA-1273.211 groups, 47% in the primary series historical control and 61% in the booster control group. Most participants were White (86% in 50 µg mRNA-1273.211, 87% in 100 µg mRNA-1273.211, 72% in the primary series historical control and 96% in the booster historical control groups), and 13%, 9%, 31% and 6% were Hispanic or Latinx in these groups, respectively. There was a higher percentage of participants who were Black or African American in the primary series historical control group (19%) compared to the groups that received 50 or 100 µg of mRNA-1273.211 (6%) or 50 µg of mRNA-1273 (3%). The percentages of participants with evidence of prior SARS-CoV-2 infection at baseline (day of the booster dose) were 1% (4/300) in the 50µg mRNA-1273.211, 2% (13/595) in the 100 µg mRNA-1273.211 groups, and 4% (6/171) in the 50 µg mRNA-1273. The median (Q1, Q3) durations between the second dose of mRNA-1273 and the booster dose were 264 (246, 276) days (50 µg mRNA-1273.211), 294 (286, 303) days (100 µg mRNA-1273.211) and 219 (199, 231) days (50 µg mRNA-1273).
Recruitment	From May 28 to June 4, 2021, and June 30 to July 15, 2021, a total of 895 participants from the Phase 3 COVE trial received a

March 2021

Recruitment	single booster dose of 50 µg (n=300) or 100 µg (n=595) mRNA-1273.211 respectively (one enrolled participant was removed from all analyses given that the participant received multiple COVID-19 vaccines outside the study, Figure 1). Participant demographic and baseline characteristics are shown in Table 1 for the two mRNA-1273.211 groups, for the primary series historical control group from the COVE trial, and for the booster historical control group[mRNA-1273 booster dose, 50 µg]) from the separate phase 2 study.
Ethics oversight	The Central IRB was Advarra, Inc., Columbia, MD. The trials have been conducted in accordance with the International Council for Technical Requirements for Registration of Pharmaceuticals for Human Use, Good Clinical Practice Guidance, and applicable government regulations. The central Institutional Review Board (Advarra, Inc, Columbia, MD) approved the protocol and consent forms. All participants provided written informed consent. The clinical trial was submitted to clinicaltrials.gov within 20 days of initiation and had no independent safety monitoring board given that it was an open-label study and the Sponsor monitored all reported adverse events.

Note that full information on the approval of the study protocol must also be provided in the manuscript.

Clinical data

Policy information about clinical studies

All manuscripts should comply with the ICMJE guidelines for publication of clinical research and a completed CONSORT checklist must be included with all submissions.

Clinical trial registration	NCT04927065
Study protocol	Online at Nature Medicine.
Data collection	Data were collected at 9 sites in the United States. Please see the Supplementary Information for the list of investigators.
Outcomes	The primary safety objective was to evaluate the safety and reactogenicity of a 50-µg or 100-µg mRNA-1273.211 administered as a single booster dose. Reactogenicity included solicited local and systemic adverse reactions (ARs) that occurred ≤7 days after the booster injection as recorded daily by participants. Unsolicited adverse events (AEs) were recorded by study sites for 28 days post-booster administration. Serious adverse events (SAEs), medically-attended AEs (MAAEs) and AEs of special interest (AESIs) were recorded by the study sites for the entire study period (~12 months).<br>There were two pre-specified immunogenicity objectives in the study. The primary objective was to demonstrate a non-inferior antibody response against the ancestral SARS-CoV-2 and the Beta variant 28 days after the booster dose of 50 or 100 µg mRNA-1273.211 candidate vaccine compared with the antibody response 28 days after the second dose of the 100-µg mRNA-1273 primary series in the historical control group based on endpoints of the antibody geometric mean titer ratio and group difference in seroresponse rates (see statistical analysis).<br>The second pre-specified immunogenicity objective was to perform non-inferiority and superiority comparisons of the antibody response of the 50-µg mRNA-1273.211 booster candidate with the antibody response of the 50-µg mRNA-1273 booster dose for the ancestral SARS-CoV-2 and for the Beta, Delta and Omicron variants based on the endpoint of the antibody geometric mean titer ratio 28 and 180 days after the booster doses (see statistical analysis). Comparison of the 100 µg mRNA-1273.211 booster vaccine candidate to the 50-µg mRNA-1273 booster dose was not part of the immunogenicity objective to compare the two booster vaccines and therefore antibody titers against Omicron and Delta after the 100 µg mRNA-1273.211 were not evaluated.
